# Syndactyly Repair

**Published:** 2013-07-15

**Authors:** Karan Chopra, Kashyap K. Tadisina, Kushal R. Patel, Devinder P. Singh

**Affiliations:** ^a^Department of Plastic and Reconstructive Surgery, Johns Hopkins School of Medicine, Baltimore, Md; ^b^Division of Plastic and Reconstructive Surgery, University of Maryland School of Medicine, Baltimore; ^c^University of Illinois, College of Medicine, Chicago

**Figure:**
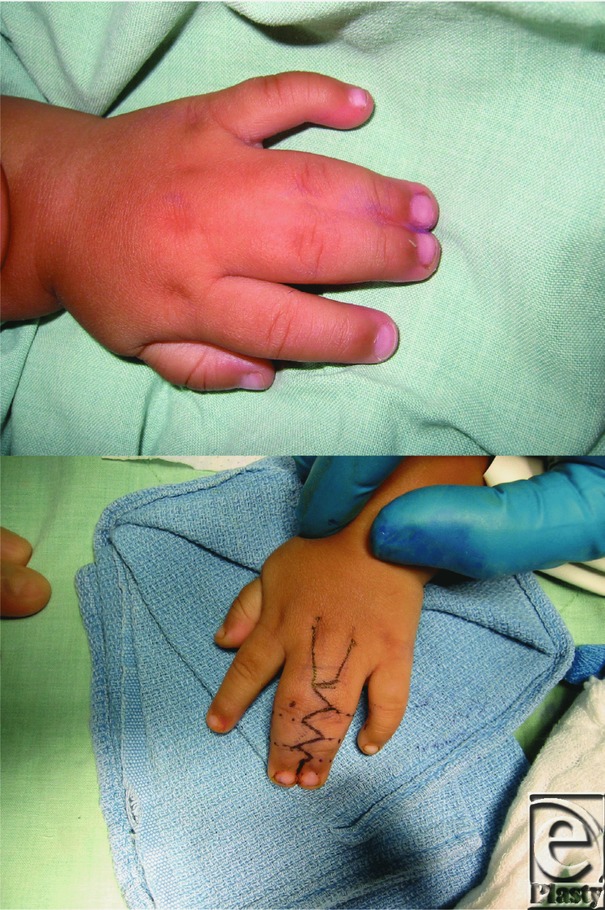

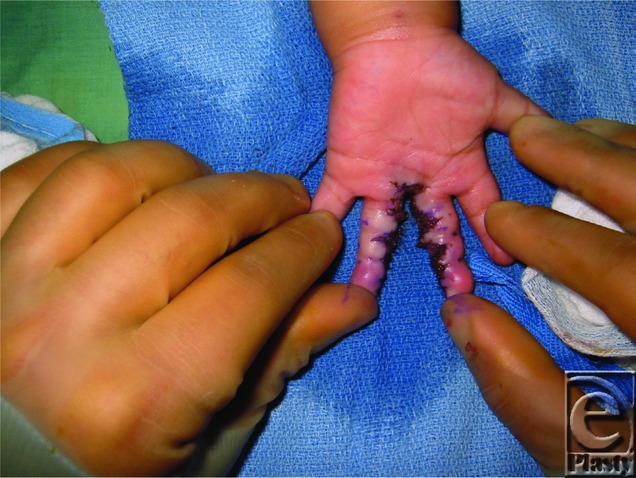


## DESCRIPTION

A 14-month-old boy presents for evaluation of fingers that are attached together as shown in the picture. The boy is diagnosed with syndactyly and prepared for surgery.

## QUESTIONS

1. **What is syndactyly? How common is it?**
2. **Discuss the embryologic basis, epidemiology, and environmental factors that can lead to syndactyly.**
3. **How is syndactyly classified?**
4. **What syndromes are most commonly associated with syndactyly?**
5. **When is the optimal time to repair syndactyly?**
6. **What are the basic principles of syndactyly repair?**
7. **What are complications associated with syndactyly repair?**
8. **Are multiple surgeries necessary for syndactyly repair?**

## DISCUSSION

Syndactyly is a congenital hand anomaly defined by adjacent digits that fail to separate during embryological development. The most common congenital hand defect, syndactyly occurs at a rate of 1 in 2000 live births, with males twice as likely to be affected. Fifty-seven percent of cases present in the third web space, and 50% of cases are bilateral.

Syndactyly occurs because of the fusion of soft tissue with or without bony fusion. The underlying defect results from the failure of interdigit mesenchymal tissue to undergo apoptosis during the seventh and eight week of gestation.^1^ While syndactyly is considered to be sporadic, an autosomal dominant pattern of inheritance has been found in those with a positive family history.^2^

Although classified anatomically, embryologically, or genetically/molecularly, syndactyly is most commonly described by the extent of webbing, degree of fusion, and the presence of duplicated skeletal parts within the interdigital web space. If the webbing extends to the fingertips, it is considered to be *complete*, while webbing that terminates more proximally is *incomplete*. A *simple* syndactyly refers to those with only soft tissue between digits, and a *complex* syndactyly refers to digits with bony or cartilaginous fusions. *Complicated* syndactyly refers to fingers with bony (ie, missing or extra phalanges), musculotendinous, and/or neurovascular abnormalities.

In patients lacking a positive family history, a higher incidence of syndactyly has been associated with maternal smoking, lower nutritional/economic status, and increased meat/egg consumption while pregnant.^3^^-^4 Syndactyly can also be found as a feature of a number of congenital syndromes, most commonly Poland, Apert, and Holt-Oram.

Syndactyly repairs are usually performed between 12 and 18 months of age to minimize scar contracture (operating too early) and deviation of the joints (operating too late). If syndactyly is complex or fingers are of significantly unequal size (ie, border digit involvement such as ring and little fingers), correction is done at 6 months of age or even earlier^5^^-^7 In repairs that involved both sides of the same digit, it is appropriate to stage repairs to protect neurovascular integrity.

With numerous successfully documented methods for reconstruction, syndactyly repair has no gold standard operation. However, all methods employ a common set of techniques that any surgeon must be comfortable with before attempting a repair. Techniques include use of full-thickness flaps, triangular/zig-zag/rectangular incisions to separate digits, preservation of vascular supply to the digit, and a meticulous approach to reconstruction with attention to preserving anatomic proportions and details.^5^^,^^6^ The most common procedures for reconstructing the web space—the most technically challenging aspect of repair—employ the use of a full-thickness skin graft from the groin region or dorsal metacarpal region, although techniques without a skin graft are gaining popularity.^5^^-^7 Historically, a full-thickness graft from the groin is used to ensure tension-free closure of open areas between separated digits. A dorsal metacarpal flap uses skin from the dorsum of the hand and extra finger skin to cover lateral defects of the fingers. One shortcoming of this technique is that it leaves a visible scar on the hand, where a graft from the groin is more conspicuous. Skin graft techniques also require intricate suturing, postoperative immobilization, and secure bandaging, the latter two of which can be cumbersome for a young active child and can lead to scar contracture or graft failure.^5^^-^8 Recently, there has been a trend toward repair of syndactyly without a skin graft. In these cases, extensive defatting is employed in the interdigit spaces. This technique, while requiring less maintenance compared to a graft, is more prone to compilations, as defatting can cause neurovascular bundle injury, reduced venous return, and/or a withered finger appearance.^5^^,^^9^

The most common acute postoperative complications of syndactyly repair are related to skin infection and necrosis, graft failure, and scar contracture related to a child's activity or lack of immobilization. These often require resurfacing with another graft and debridement. Long-term complications include web creeping, skin graft dysmaturation, keloid formation, and joint instability/deformity. “Web creeping” is the recurrence of abnormal webbing between digits that occurs because of scar contracture at the base of attachment or along incision lines.^8^ The use of full thickness over partial thickness grafts, zig zag incisions, and early release of border digits are techniques used to prevent this.^8^ Skin grafts that become hyper pigmented or are taken from areas that result in hirsute hair growth can be replaced or esthetically maintained. Keloid formation has been reported to occur in 1% to 2% of cases.^5^ In complicated cases of syndactyly, reduced mobility, skeletal deformities, and joint instability may occur, and arthrodesis is considered to remedy issues once the child is skeletally mature.^6^^,^^9^

Follow-up surgeries for syndactyly are usually not necessary. However, if severe contractures develop, a second graft or release will need to be done, and secondary operative rate has been reported to be around 10%.^5^ Complicated cases such as polysyndactyly and those with postoperative complications are more frequently operated on secondarily.^6^
